# The effects of pentoxifylline on skeletal muscle contractility and neuromuscular transmission during hypoxia

**DOI:** 10.4103/0253-7613.58509

**Published:** 2009-10

**Authors:** Fatma Simsek-Duran, Mert Ertunc, Rustu Onur

**Affiliations:** Department of Physiology, Faculty of Medicine, Hacettepe University, 06100 Sihhiye, Ankara, Turkey; 1 Department of Pharmacology, Faculty of Medicine, Hacettepe University, 06100 Sihhiye, Ankara, Turkey

**Keywords:** Contractility, hypoxia, neuromuscular transmission, pentoxifylline

## Abstract

**Objectives:**

The objective of this study was to investigate the effects of pentoxifylline (PTX), a drug that is mainly used for indications related to tissue hypoxia, on hypoxia-induced inhibition of skeletal muscle contractility and neuromuscular transmission in mice. We hypothesized that chronic PTX treatment alters skeletal muscle contractility and hypoxia-induced dysfunction.

**Materials and Methods:**

Mice were treated with 50 mg/kg PTX or saline intraperitoneally for a week. Following ether anesthesia, diaphragm muscles were removed; isometric muscle contractions and action potentials were recorded. Time to reach neuromuscular blockade and the rate of recovery of muscle contractility were assessed during hypoxia and re-oxygenation.

**Results:**

The PTX group displayed 90% greater twitch amplitudes (*P* < 0.01). Hypoxia depressed twitch contractions and caused neuromuscular blockade in both groups. However, neuromuscular blockade occurred earlier in PTX-treated animals (*P* < 0.05). Muscle contractures developed during hypoxia were more pronounced in the PTX group (*P* < 0.05). Re-oxygenation reduced contracture and indirect muscle contractions resumed. The rate of recovery of contractions was faster (*P* < 0.05) and the amplitude of contractions was greater (*P* < 0.01) in the PTX group. PTX treatment increased amplitude (*P* < 0.05) and shortened action potential (*P* < 0.05) without altering resting membrane potential, excitation threshold, and neurotransmitter release.

**Conclusion:**

Chronic PTX treatment increases diaphragm contractility, but amplifies hypoxia-induced contractile dysfunction in mice. These results may implicate important clinical consequences for clinical usage of PTX in hypoxia-related conditions.

## Introduction

Hypoxia inhibits skeletal muscle contractility and causes neuromuscular blockade. Intracellular mechanisms of hypoxia-induced damage in skeletal muscle have not been fully elucidated. Impaired calcium (Ca^2+^) buffering and decreased adenosine-5'-triphosphate (ATP) production in myocytes are known to participate in hypoxia-induced impairment of muscle function.[1]

Pentoxifylline (PTX), a xanthine-derived phosphodiesterase (PDE) inhibitor, is known to increase intracellular cyclic-adenosine monophosphate (cAMP) levels.[2] An increase in cAMP levels in muscle fibers results in the activation of protein kinase A (PKA) and facilitates synaptic transmission in the mammalian neuromuscular junction (NMJ).[3] Recent data showed that PTX administration decreases plasma levels of pro-inflammatory cytokines, such as tumor necrosis factor alpha(TNF-α), interleukin (IL)-1 β, IL-10,[4] and down-regulates their production.[5] Recently, cytokines have been shown to impair contractile response in the skeletal muscle probably by affecting the nitrergic pathway or nitric oxide (NO) production.[6,7]

PTX exerts beneficial effects on the treatment of peripheral circulatory insufficiency. A recent study has demonstrated that PTX attenuates ischemia reperfusion injury in the skeletal muscle and other tissues by decreasing neutrophil adhesion to endothelial cells, reactive oxygen species production, and platelet activation.[8] However, the effects of PTX treatment on muscle contractility and on hypoxia-induced contractile dysfunction have not been studied. This study was designed to evaluate the effects of chronic PTX treatment on the contractile properties of the skeletal muscle and hypoxia-induced contractile impairment. We hypothesized that chronic PTX treatment alters skeletal muscle contractility and hypoxia-induced dysfunction.

## Materials and Methods

Mice, weighing between 30 and 35 g, were obtained from the Hacettepe University, Department of Pharmacology Animal Breeding Unit. Animals were housed in communal cages with food and water available *ad libitum*, and exposed to a 12 h light/dark cycle with the room temperature maintained at 21°C.

The procedures in the study were approved by Hacettepe University Animal Care and Use Ethics Committee, Ankara, Turkey, approval numbers 2001/08-3. Experiments were conducted in accordance with the Animal Care Guidelines of Hacettepe University.

Experiments were performed in two groups of mice, PTX, and control. In the PTX group, animals were treated with PTX (50 mg/ kg, i.p., n = 12) for a week and the control animals received saline (n = 6). Animals were anesthetized with ether and phrenic nerves attached to the diaphragm muscles were removed. Ringer's solution had the following composition (in mm): NaCl, 135; KCl, 5; MgCl_2_, 1; CaCl_2_, 2; NaHCO_3_, 15; Na_2_HPO_4_, 1; and glucose, 11. Muscle preparations were placed vertically in a 50 ml organ bath at 37°C and gassed with a mixture of 95% oxygen (O_2_) and 5% carbon dioxide (CO_2_). After applying 1-1.5 g resting tension, isometric muscle contractions were recorded via force displacement transducers (FT03, Grass Instruments, Quincy, MA) on a polygraph (Model 79C Grass Instruments, Quincy, MA). Phrenic nerve and diaphragm muscles were stimulated by rectangular electrical pulses via platinum electrodes connected to a stimulator (Model S88 Grass Instruments, Quincy, MA) and a stimulus isolation unit (SIU5 Grass Instruments, Quincy, MA). A pair of platinum electrodes was placed vertically to both sides of the muscle fibers for direct stimulation. The phrenic nerve was placed between two parallel platinum ring electrodes, which were placed in a polyethylene tubing of 3 mm diameter. Contraction amplitudes of muscles were normalized and expressed as g/g of wet tissue. In order to induce hypoxia, 95% nitrogen (N_2_) + 5% carbon dioxide (CO_2_) gas mixture was applied. Isometric muscle contractions, time to reach complete neuromuscular blockade during hypoxia, and the rate of recovery of muscle contractility upon re-oxygenation were determined. Electrophysiological experiments were made by using conventional microelectrode techniques. Muscle preparations were placed on perspex plates covered with Sylgard, the thoracic side on top and were perfused with Ringer's solution 1-2 ml/min at 22 ± 1°C. Muscle preparations were illuminated from the bottom of the organ bath and were observed by a stereomicroscope (Bausch and Lomb). Resting membrane potentials (RMPs), miniature end plate potentials (MEPP), and action potentials (APs) were recorded with borosilicate glass microelectrodes having 10-30 MΩ of input resistances filled with 3 mol/l KCl. Signals were amplified by Axoclamp 2A (Axon instruments, Foster city, CA, USA) amplifier and displayed on a digital storage oscilloscope (Hitachi VC-6524, Hitachi Denshi, Tokyo, Japan). Amplitude and frequency of MEPPs, threshold level, overshoot, amplitude, and half-rise and half-decay times of APs were evaluated and digitized by Powerlab 8/P (ADI Instruments, Pty. Ltd., Castle Hill, Australia) and Scope 3.2 and Chart 4.0 software programs were used to analyze data.

The following drugs were used: Pentoxifylline (Trental, Roche Istanbul, Turkey) and caffeine (Sigma Chemical Co., MO, and USA).

All data were expressed as mean ± SEM. Comparison of the groups was done by the Mann-Whitney *U*-test or two-way ANOVA followed by Bonferroni's post *hoc* test. Statistical significance was defined as *P* < 0.05.

## Results

**Figure 1:**
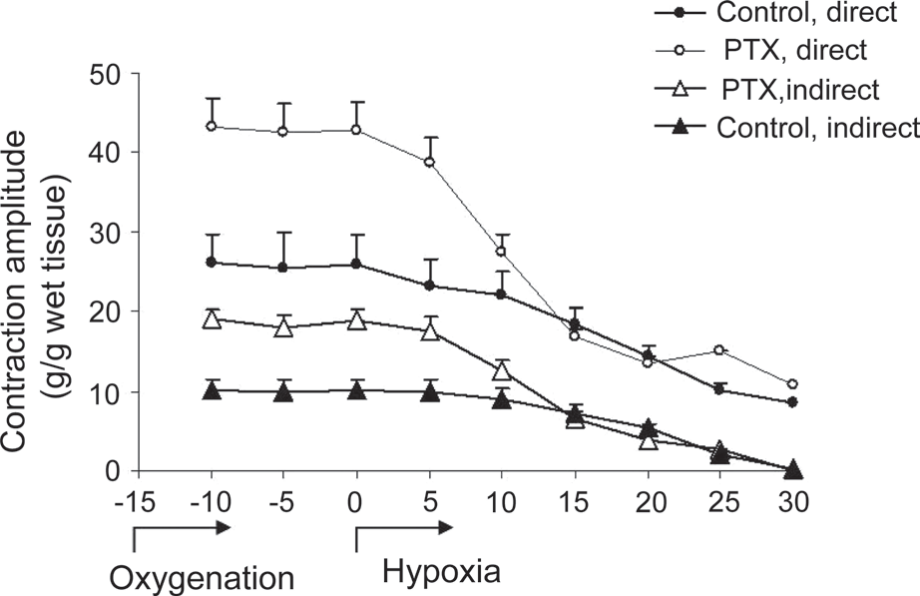
Effects of hypoxia on diaphragm muscles obtained from PTX-treated mice. After 15 min of the oxygenation period, hypoxia was instated by 95% N_2_ + 5% CO_2_ until neuromuscular blockade occurred (control, n = 6 and PTX, n = 12, *P < 0.01); *P* < 0.01, two-way ANOVA, *post hoc* Bonferroni

PTX treatment increased contractility of the diaphragm muscles. Amplitudes of direct and indirect isometric muscle contractions in the PTX-treated group were greater than controls. Diaphragm muscles prepared from the control animals had twitch contraction amplitudes of 22.2 ± 4.5 g/g and 10.0 ± 1.4 g/g, upon direct and indirect stimulation, respectively. Muscles obtained from PTX-treated animals displayed about 90% greater contraction amplitudes for both types of stimulation [Figure 1, *P* < 0.01].

**Figure 2:**
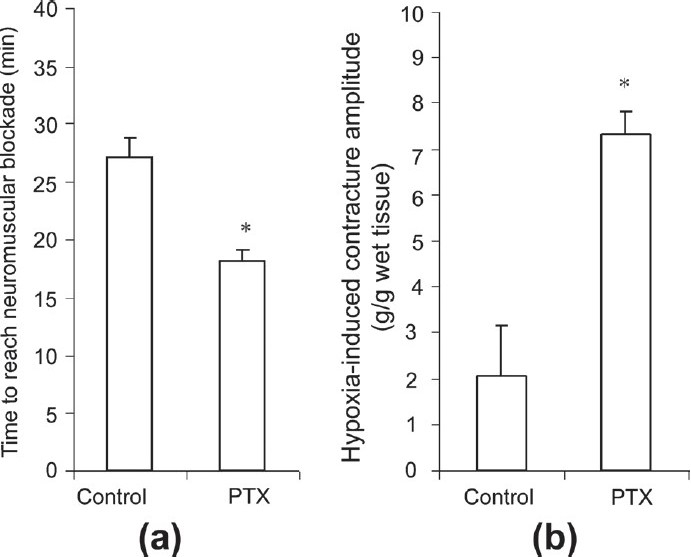
Effects of hypoxia on time to reach complete neuromuscular blockade (a) and contracture development (b) in muscles of PTX-treated mice (control, n = 6, PTX, n = 12, *P < 0.05); *P* < 0.05, the Mann-Whitney *U*-test

Hypoxia inhibited muscle contractions, caused neuromuscular blockade, and induced a reversible muscle contracture in both groups. Effects of hypoxia were more pronounced in the PTX group. Complete neuromuscular blockade occurred 28.5 ± 5.7 min after the commencement of hypoxia, in the control group. However, in the PTX group, this effect occurred 22.5 ± 6 min later [Figure 2a, *P* < 0.05]. The hypoxia-induced contracture amplitude in the PTX group was significantly greater than the controls, 7.3 ± 0.5 and 2.1 ± 1.1 g/g in the PTX-treated and controls, respectively [Figure 2b, *P* < 0.05].

**Figure 3:**
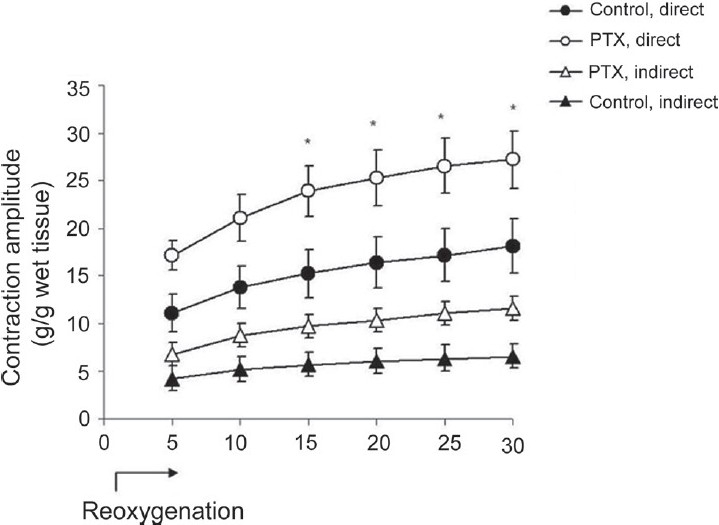
Effects of re-oxygenation in PTX-treated diaphragm muscles. Contractile recovery from hypoxia was investigated for 30 min in muscle strips in the presence of 95% O_2_ + 5% CO_2_ (control, n = 6 and PTX, n = 12, *P < 0.01); *P* < 0.01, two-way ANOVA, *post hoc* Bonferroni

**Figure 4:**
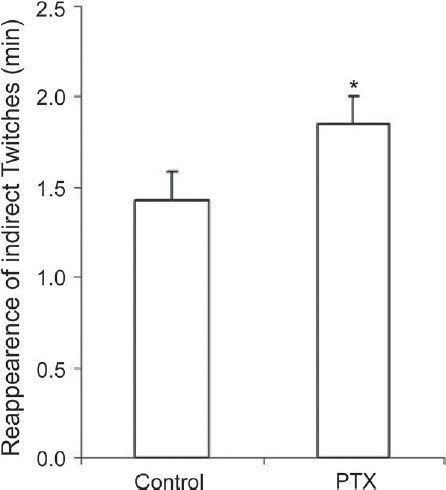
Reappearance of indirect muscle twitches in PTX-treated mice during re-oxygenation (control, n = 6, PTX-treated, n = 12, *P < 0.05); *P* < 0.05, the Mann-Whitney *U*-test

Re-oxygenation was instated in all preparations after completion of neuromuscular transmission failure. During 30 min of re-oxygenation, indirect muscle twitches reappeared, hypoxic contracture returned to the baseline, and muscle contractility partially recovered [Figure 3]. Indirect muscle twitches reappeared 1.4 ± 0.2 min after re-oxygenation in the control group and 1.8 ± 0.2 min later in the PTX group [*P* < 0.05, Figure 4]. In this period, amplitudes of direct and indirect contractions of the diaphragm muscles prepared from the PTX-treated mice were significantly greater [*P* < 0.01, Figure 3]. However, the rate of recovery of muscle contractility upon re-oxygenation was slower in the PTX group. At the end of the re-oxygenation period, direct muscle contractions in the control group recovered to 82% of the baseline values and indirect contractions recovered to 65%. These ratios in the PTX group were 63% for direct contractions and 61% for indirect contractions.

PTX treatment did not appreciably alter RMP and spontaneous neurotransmitter release in the mouse diaphragm muscles. Control and PTX-treated muscles had mean MEPP frequencies of 0.96 ± 0.1 per s and 0.99 ± 0.09 per s, and amplitudes of 1.93 ± 0.11 mV and 1.86 ± 0.09 mV, respectively. RMP of control muscles was 75.3 0.3 mV and did not alter in skeletal muscles of PTX-treated mice (74.8 0.4 mV).

**Figure 5:**
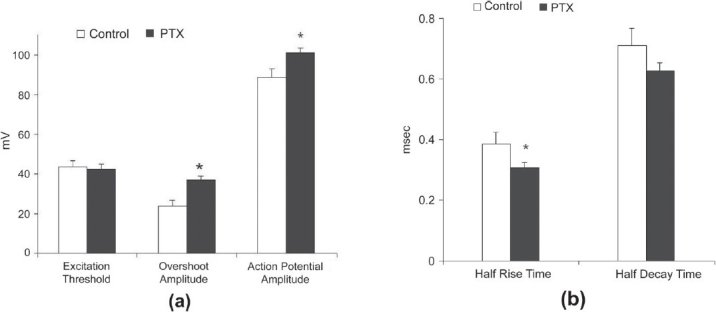
Effects of PTX treatment on excitation threshold overshoot and action potential amplitudes; (a) (control, n = 4, 40 muscle fibers; PTX-treated n = 7, 40 muscle fibers) half rise and half decay times recorded from diaphragm muscles by indirect stimulation; (b) (control, n = 4 muscles and 59 fibers; PTX-treated, n = 12 muscles and 40 fibers, *P < 0.05); *P* < 0.05, the Mann-Whitney *U*-test

PTX treatment significantly altered AP configuration of the diaphragm muscles without changing threshold for excitation. APs recorded from PTX-treated animals were increased in amplitude and had a faster time course. APs of these muscles were about 13 mV greater than that of controls [Figure 5a, *P* < 0.05]. Half-rise times of the APs of the PTX-treated muscles were significantly [Figure 5b, *P* < 0.05] shorter than that of control muscles and found to be 0.30 ± 0.04 ms and 0.38 ± 0.07 ms, respectively. Half-decay times of the PTX-treated group were also shorter than the control group (0.60 ± 0.07 ms and 0.71± 0.05 ms, respectively); however, this difference did not reach statistical significance.

## Discussion

This study demonstrated that chronic PTX administration increased twitch and AP amplitudes of diaphragm muscle, in mice. PTX treatment shortened time course of muscle APs without altering RMP, excitation threshold, and spontaneous neurotransmitter release. Contractile impairment in hypoxia was more pronounced with prolonged recovery upon re-oxygenation.

One of the mechanisms involved in the increase in contractility observed in PTX-treated animals appears to be related to PDE inhibition, which alters mechanisms related to cytosolic Ca^2+^ handling.[9,10] In our study, PTX-treated diaphragm muscles did not display alteration of KCl and caffeine contractures, implying that PTX at this dose level did not change depolarization-contraction coupling and Ca^2+^ release from the sarcoplasmic reticulum (SR).

Regulatory effects of PTX on cytokine expression may contribute to increased skeletal muscle contractility. PTX decreases the expression of TNF-α in various tissues, including the skeletal muscle.[5] PTX also inhibits cellular TNF-α synthesis and attenuates toxic effects of TNF-α.[4,11] It is shown that cytokines, predominantly TNF-α have a prominent role in skeletal muscle contractile dysfunction in these cytokine-related pathologies.[12] In a recent study, Alloatti *et al*. showed that TNF-α reduced contractility and shifted the force-frequency relationship to the right in guinea pig extensor digitorum longus muscle. They also found that the TNF-α-induced effect is mediated by NO production, which is induced by a platelet-activating factor.[13] In another study, TNF-α induced reduction of contractile force in cardiac myocytes was found to be dependent on excessive NO production.[6] NO production impairs contractile response in the skeletal muscle by acting on intracellular Ca^2+^ transients, SR Ca^2+^ ATP-ase expression, and/or Ca^2+^ release channel.[14] We found that muscles obtained from PTX-treated mice had greater twitch amplitudes in normoxic conditions, however, in the hypoxic state; the contractility of PTX-treated muscles was lower than that of controls. This may be related to the diverse effects of PTX on basal and hypoxia-induced local expression of cytokines, specifically, to TNF-α and IL-6 which have recently been shown to induce various effects on skeletal muscle protein metabolism, proliferation, NO production, and contractility.[15] We also found that in the re-oxygenation period, amplitudes of direct and indirect twitch contractions of PTX-treated muscles were greater, indicating a more efficient recovery process. Greater contractile responses obtained in our study in PTX-treated muscles may be explained in part, by NOS inhibition and/or decreased NO production.

Detrimental effects of hypoxia on contractility were increased in muscles obtained from PTX-treated animals. This is evidenced by the findings that hypoxia-induced twitch amplitude inhibition and contracture were greater in PTX-treated muscles. Also recovery upon re-oxygenation was delayed in these muscles. These effects may be due to increased Ca^2+^ influx after PDE inhibition by PTX treatment. Excessive amounts of cytosolic Ca^2+^ is deleterious, and it has been shown that elevated Ca^2+^ induces disruption of the excitation-contraction (E-C) coupling.[16] Increased intracellular Ca^2+^ concentration activates proteases and phospholipases, which contribute to the damage in the mitochondria and SR.[17–19] Damage of these organelles eventually causes progression of the deterioration by further increasing intracellular Ca^2+^ levels. Hypoxia is one of the triggering events initiating this vicious cycle by decreased ATP content to maintain intracellular Ca^2+^ balance.[20]

Our data showed that chronic PTX treatment did not alter RMP of diaphragm muscle and spontaneous neurotransmitter release at the neuromuscular junction. However, PTX treatment significantly increased muscle AP amplitudes and shortened their time course. The reduction in time course was found to be more prominent in the rising phase, implying increased Na^+^ channel activity. PDE inhibitors are reported to alter electrical activity of cardiac and skeletal muscles. Toborinone, a PDE inhibitor by increasing cAMP concentration increased contractility and prolonged AP duration in cardiac myocytes by blocking the delayed rectifier currents.[21] We observed an increase in skeletal muscle contractility concomitant with reduction in AP duration. This difference in AP duration may be due to different types of muscles and drugs used. Recently, Gonzalez-Serratos *et al*. showed that a different PDE inhibitor, LASSBio-294, did not alter RMP and APs in frog sartorius muscles; however it improved contractility and reduced muscle fatigue.[22]

## Conclusion

Chronic PTX treatment increases skeletal muscle contractility without altering spontaneous acetylcholine release, but does not improve hypoxia-induced skeletal muscle dysfunction. These results may implicate important clinical consequences for PTX, a drug that is mainly used for indications related to tissue hypoxia.
